# Scotch Physicians and Surgeons

**Published:** 1888-02-04

**Authors:** C. G. Furley


### Scotch Physicians and Surgeons.

By C. G. Furley.

*(Continued from p. 247.)*

### SIR JAMES Y. SIMPSON.

Of the revolution brought about by chloroform in every branch of surgery it is needless now to speak; but it is worth noting how the news was received forty years ago.

First it should be observed that Simpson never claimed to have "invented" or "discovered" chloroform. Various attempts have been made and articles written to take from him an honour which he never sought. "Chloroform," he says, "was first discovered and described at nearly the same time by Soubeiran (1831), and Liebig (1832); its composition was first accurately ascertained by the distinguished French chemist, Dumas, in 1835." The distinction which belongs to Simpson—and surely it is a most honourable one—was that after testing its qualities by various experiments, he introduced it into practice, and gave the boon of immunity from pain to the suffering world. "They"—the original discoverers—"had no idea that the substance to which they called the attention of their chemical brethren could or would be turned to any *practical* purpose, or that it possessed any physiological or therapeutic effect upon the animal economy. . . . Here, then, we have a substance which in the first instance was merely interesting as a matter of scientific economy and research, becoming rapidly an object of intense importance as an agent by which human suffering may be annulled and abolished under some of the most trying circumstances in which human nature is ever placed."

Naturally, the paper which contained these remarks created intense excitement, and the fame of it soon spreading beyond the society before whose members it had been read, it was reprinted as a pamphlet, entitled "Account of a New Anæsthetic Agent, as a Substitute for Sulphuric Ether in Surgery and Midwifery," and was dedicated to Dumas, the great French chemist, who had described its composition. Four thousand copies of this were sold in a few days, and the news of the anæsthetic spread like wildfire through the country. Every incident connected with the discovery of its therapeutic action was seized upon, and eagerly quoted. Some of these were interesting enough. Simpson told how he had had a specimen of chroloform prepared, but, judging from the heavy appearance of the liquid that it would not serve the purpose he desired, he neglected to make any experiment with it. One evening, however, coming into his house with his two assistants, Drs. Thomas Keith and Matthews Duncan (both names of note to-day in the world of obstetric medicine), his hand fell upon the bottle of chloroform while he was searching for another substance. He suggested that they should make a trial of its qualities, and with a rashness which medical men never display except when they themselves are the subjects of their experiments, they all inhaled it simultaneously, and with such excellent result that in a minute or two they were all lying prone and unconscious "under the table." Mrs. Simpson discovered them first, with what feelings of anxiety need not be described.

But while the public at large were singing Simpson's praises, while patients who had themselves undergone the agony of surgical operations were thanking heaven that others need not suffer as they had done, there arose dissentient voices, some saying that the discovery of chloroform was not new; some that the new anæsthetic was not safe; some—oh! miraculous eccentricity of the human mind!—that its use was immoral! It is sad to think that these protests came chiefly from professional brethren. Those who claimed to have anticipated Simpson's discovery were mostly men who confounded it with ether, a thing which had been used for some time both in England and America; and were easily disposed of. Many timid practitioners hesitated to employ it, except in extreme cases, lest a death occurring while the patient was in a state of anæsthesia should bring upon them an accusation of murder; but as they became familiar with the best method of giving it and the precautions that were necessary during its application, the scruples of these nervous individuals died away. Those who objected to chloroform on ethical grounds were the most persistent, as in the eyes of a later generation they are the most remarkable of the three classes.

It is certainly amusing to read now the solemn protests of disapproval which many worthy people launched at Simpson and his anaesthetic. Judging by their utterances, one would think that pain was ordained as the primal blessing rather than the primal curse. If the dictum of Shakespeare be true—and our experience tends to verify it—that "there was never yet philosopher that could endure the toothache patiently," we are forced to assume that these estimable stoics had never in their lives suffered from any worse ailment than a cold in the head. A certain Dr. P., of Liverpool, wrote an article, entitled "A Branch of *Medical Ethics*," in which he left the well-trodden path of the medical practitioner to mount the orator's rostrum. "What right have we, even as men," he asks, "to say to our brother man, 'Sacrifice thy manhood; let go thy hold upon that noble capacity of thought and reason with which thy God hath endowed thee, and become a trembling coward before the presence of mere bodily pain.'"

It is to be hoped that Dr. P. had convinced himself of the truth and justice of his criticisms, for otherwise his next remarks must be regarded as grievously irreverent. If man, as man only, he argued, was bound to accept pain, Christians were much more bound to receive it gladly; and the crucified Redeemer was dragged into the controversy to prove the critic's point. Simpson took the trouble to argue this point seriously, and showed that Biblical research tended to prove that the spongeful of "gall mingled with vinegar" which was given to the Saviour was a stupefying drink habitually offered to criminals to lessen their sufferings; was indeed akin to those anæsthetics of early times to which Dioscorides and other ancient practitioners allude, but whose exact nature and composition tradition has failed to transmit. To us so much explanation seems wholly unnecessary. To accept inevitable pain bravely and to avoid all other are, in our eyes, things equally to be commended; we have—most of us, at least—got beyond the mediæval idea that suffering is, in itself, meritorious.

Finally, Dr. P., returning from his flight into regions "where angels fear to tread," came down to the lower realms of imagination, and prophesied that in many cases death would ensue from the use of chloroform, and insanity in the rest. He did not bring forward any proofs of the accuracy of his predictions. What need? Can anything be more impressive than a judicious vagueness? It was not even in so many words that Lord Burleigh (in the *Critic*) foretold the destruction of the English fleet; but merely by a solemn shake of the head. As a matter of historical fact, England destroyed the Armada; but the head-shake was awe-inspiring all the same; and the Cassandra-like warnings of Simpson's opponents, unfounded as they were, served for a time to make one or two people refuse to profit by the precious boon of chloroform.

Even those who thought that—for the convenience of the operator—chloroform might advisably be adopted for surgery were much averse from its being employed in midwifery. The curse of Eve was sacred; and men who did not mind avoiding pain themselves felt that their deepest feelings would be outraged if a woman escaped a single pang. It all reads like a solemn farce now; but it was a serious reality at the time, and Simpson's discovery was regarded by many as tending to undermine religion and morality.

But this could not go on long. "The patients themselves will force it into use," said Dr. Simpson, and the remark being repeated was made the occasion of a new outcry. Was the patient to obey the doctor, or the doctor the patient? This was preaching a gospel of rebellion! It seemed that the power which some practitioners of the healing art valued most was the power of inflicting pain.

To quench the alarm, Simpson wrote and published full descriptions of his anæsthetic, its composition and action, as well as the reasons which, in his opinion, justified him in using it, and encouraging its use. This brought out a new war-cry; he "was telling the public of matters which ought to be known only to the profession." What importance this accusation may carry to the lay mind—indeed, to any mind that can see an inch beyond the selfish advantage of personal monopoly—it is difficult to find out. It sounds vague, to say the least of it. Surely in 1848 doctors should have got beyond being "mystery men;" they should have dared to tell their patients why such-and-such a drug was useful, and what would be its immediate and subsequent effects. Men and women were not then so childish as not to be impressed by a treatment because they could comprehend it; and indeed, even with children, an appeal to their intelligence, an explanation why the "nasty physic" should be taken, will often win them to submit to necessary disagreeables more readily than irrational persistence and force. The public has a moral right to understand the laws of health, the phenomena of disease, and the action of remedies, if it chooses to seek instruction in these matters; they immediately concern the individual and general well-being, and no man regards his doctor with less respect because he understands the reasons of his treatment. Rather is he likely to appreciate his services the more.

But on the whole it is needless to say much about the reasons why the introduction of chloroform met with such opposition. It is probable that they may all be condensed into two words—professional jealousy! The spectacle has been seen, both before and since those days, of old and stationary, if not retrograde, individuals and institutions resenting as a personal injury being passed by juniors possessed of greater energy and enlightenment. But they never confess to such a petty feeling as jealousy; they always disapprove on public grounds and from the highest moral principles. It was not strange that, vexed by these gnats and wasps of the medical world, a man of Simpson's impetuous and honest temperament should have launched some scornful phrases at them. If thereby he made some enemies, they were probably such as would have injured him and his cause more had they been friends.

And meanwhile chloroform, and the man who gave it to the world, went on their way and prospered. The new drug was soon to be obtained in every town in the kingdom, and had found its way into the villages, where it was dispensed only under the severest restrictions, as Dr. Simpson himself once discovered to his cost.

While on an archæological excursion with one of his sons, the boy was attacked with toothache. His father went to the druggist's shop in a village near, and asked the dispenser, who was of the fairer sex, for a dose of chloroform to soothe the pain. "Na, na," she replied to the request of the man who knew more about chloroform than any in the land—"Na, na; we dinna sell chloroform to folk that kens naething about it."

Although chloroform was so well received, though it was used everywhere, from the palace to the peasant's hut, and though the danger attending its employment was, with proper care, of the very slightest, Simpson himself was always seeking for another and superior anæsthetic. The experiments into which he was thus led were sometimes unfortunate in their results. On one occasion he was found lying helpless, apparently, but not really, unconscious, by one of his women-servants. She called the butler to aid in restoring her master to consciousness, and Simpson heard the latter—a firm believer in the virtues of chloroform—say: "He's aye trying to find out something else; and he's just a big fule, for he'll never get onything better than the chlory." It is said that this candid opinion acted as an admirable restorative, and the doctor roused himself from his swoon to have a hearty laugh at his retainer's words.

(*To be continued.*)

